# Comparison of Vitamin D, Neurofeedback, and Neurofeedback Combined with Vitamin D Supplementation in Children with Attention-Deficit/Hyperactivity Disorder

**DOI:** 10.34172/aim.2022.47

**Published:** 2022-05-01

**Authors:** Masoud Rahmani, Azadeh Mahvelati, Amir Hossein Farajinia, Shima Shahyad, Mojtaba Khaksarian, Roghieh Nooripour, Saba Hassanvandi

**Affiliations:** ^1^School of Medicine and Surgery, University of Milano-Bicocca, Milano, Italy; ^2^Department of Counseling, Imam Reza International University, Mashhad, Iran; ^3^Department of Psychology, Faculty of Humanistic Sciences, Khorramabad Branch, Islamic Azad University, Khorram Abad, Iran; ^4^Neuroscience Research Center, Baqiyatallah University of Medical Sciences, Tehran, Iran; ^5^Lorestan University of Medical Sciences, Khorramabad, Iran; ^6^Department of Counseling, Faculty of Education and Psychology, Alzahra University, Tehran, Iran

**Keywords:** Attention-deficit/hyperactivity disorder, Brain waves, Combined treatment, Neurofeedback, Supplementation, Vitamin D

## Abstract

**Background:**

Nowadays, some treatments such as neurofeedback and Vitamin D Supplementation are of great importance in the treatment of attention-deficit/hyperactivity disorder (ADHD). To determine the efficacy of the combined treatment, the present trial was conducted to investigate the effectiveness of each one of them with combined neurofeedback and vitamin D supplementation in the reduction of ADHD symptom in children suffering from this disorder.

**Methods:**

In this study from March 2020 to June 2020, we enrolled a total of 120 patients (6-15 years old) who were referred to the Mehr psychiatric hospital (affiliated to Lorestan University of Medical Sciences). Patients were then randomly categorized into three experimental groups and one control group. The first, the second, and the third experimental groups consumed vitamin D pearl, neurofeedback combined with vitamin D, and neurofeedback for 12 weeks, respectively. The control group was given no treatment. Vitamin D serum level was evaluated at baseline, 4, 8, and 12 weeks in all participants. For data collection, the Parent Attention-Deficit/Hyperactivity Disorder Rating Scale-IV (ADHD-RS-IV) was applied. The obtained information was analyzed using repeated measure variance analysis.

**Results:**

The mean scores were significantly different across the groups. Repeated measure variance analysis showed that the mean score was lower in the combined group in comparison with the other three groups (*P*<0.05).

**Conclusion:**

Combined treatment could be considered as more effective compared to separate treatments. In addition, in this study, by applying the combined intervention, the duration of treatment decreased significantly.

## Introduction

 Nowadays, a variety of treatments are studied to treat the attention-deficit/hyperactivity disorder (ADHD). Therapeutically, pharmacological intervention is the most popular treatment for ADHD. Accordingly, in Iran, the most famous drug is methylphenidate and atomoxetine.

 The main signs of ADHD are an obvious trend of inattention, hyperactivity, impulsivity, and distraction.^1^ It is worth highlighting that ADHD symptoms often coincide with comorbid symptoms such as oppositional behaviors, mood problems, aggression, impaired social functioning and anxiety.^2^ Up to now, pathological findings have shown that both frontal lobe and right hemisphere hypometabolism in patients with ADHD play important roles in its manifestations compared with normal controls. Accordingly, the frontal lobes, especially the right frontal lobe, are essential for the maintenance of attention in this disorder.^3^

 Studies performed on the possible causes of this disorder have indicated that neurodevelopmental genes and the deficiency of some nutritional supplements are effective in the onset of ADHD symptoms.^4^ Evidence has shown that compared with healthy controls, serum vitamin D receptor levels are considerably lower in children with ADHD.^5,6^ Notably, vitamin D is a fat-soluble material that plays a key role in calcium (Ca) and phos­phorus (P) homeostasis, with hormone-like functions.^6^ A meta-analysis showed that there is a negative connection between 25-hydroxyvitamin D level [25 (OH) D] and diagnosis of ADHD in young people with ADHD. In another study,^7^ the amount of vitamin D was investigated in patients suffering from ADHD. For this purpose, a total of 60 patients with ADHD and 30 healthy controls were investigated in their study. The results showed that the level of serum 25-OH-vitamin D was substantially lower in the experimental sample than healthy controls.

 The use of supplementation for therapeutic aims has been approved by previous studies. It has been demonstrated that serotonin synthesis and action are controlled by vitamin D and the omega-3 fatty acids in some disorders such as ADHD, bipolar disorder, psychotic disorders, and aggressive behavior.^8^ Hence, vitamin D could be considered to be more effective than other supplements. Some studies have provided evidence regarding the relationship between 25 (OH) D levels in blood of pregnant mothers or newborn babies at nascency and neurodevelopmental consequences, including some problems in brain activities, motor dysfunction, verbal deficiencies, and abnormal behaviors.^9^ This research and other similar clinical studies have shown that at some stage in being pregnant or sometimes in the newborn period, the blood level of vitamin D is lower in some disorders like ADHD.

 Many attempts have been made to discover more effective treatments that are less dangerous for neurological conditions. In this regard, neurofeedback is one of the newest treatments known for ADHD. Neurofeedback is a new, popular and noninvasive intervention for treatment of many nervous system dysfunctions. Other conditions in which neurofeedback is used are as follows: ADHD,^10^ learning disabilities,^11^ strokes,^12^ head injury,^13^ insomnia,^14^ depression,^15^ obsessive–compulsive disorder,^16^ and drug addiction.^17^ The underlying assumption is that brain waves reflect neural functions completely, and that regulating brain waves may promote neural system performance, which subsequently leads to reduction of ADHD symptom and promotion of cognitive performance.^18^ One study performed a comparison between the effect of pharmacotherapy and neurofeedback on oral health of students with ADHD. Finally, the result showed that unstimulated salivary flow of children with ADHD who used Ritalin was significantly lower than the children with ADHD in the neurofeedback group. Also, neurofeedback intervention is preferable to Ritalin for children with ADHD in terms of maintaining their oral health.^19^ Moreover, the results indicated that the combined (ADHD-C) and impulsive/hyperactive (ADHD-HI) would achieve greater improvements than inattentive (ADHD-I).

 The effectiveness of each of these techniques such as neurofeedback and the use of supplementation like vitamin D, has been studied in various studies. Besides, comparative studies have also been conducted in this field. For example, this gap has been investigated by some studies.^20^ The results have shown that both treatments are useful in various studies, but no clear superiority has been shown yet. In addition, other studies investigated possible treatments for achieving the least dangerous and fastest possible treatments.^21^

 Patients with ADHD not only experience educational problems, but they are also at increased risk for antisocial behavior and substance abuse, which burden every health system.^22^ Given the fact that ADHD is a complicated varying condition, it has been assumed that treating with one approved supplementation is highly unlikely to increase dangerous or complex side effects.^23^ So, it seems that using the proved combined treatments could increase therapeutic outcomes. Moreover, it appears that combined treatments can be more efficient in decreasing ADHD symptoms than separate treatments. Considering various clinical trials that have investigated the effectiveness of these two treatments alone, this study attempts to compare the combined treatment and separate treatment as well as determining the difference in their effectiveness.

## Materials and Methods

###  Study Design and Setting

 This randomized, double-blind with parallel-group clinical trial was conducted over three months between March 2020 to June 2020 at the Mehr psychiatric hospital (affiliated to Lorestan University of Medical Sciences). Regarding ethical issues, after thorough explanation of the whole process and the purpose of the trial to the participants, a written informed consent was obtained from the parents of all children. Moreover, the authors provided complete explanation to each patient’s parents to reassure them that they are completely free to withdraw from the trial at any time they wished.

###  Sample Size

**Figure 1:**
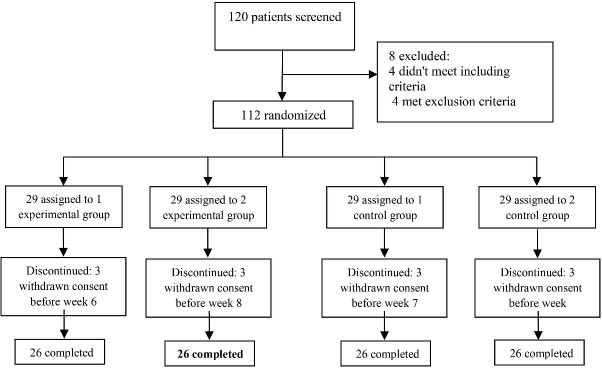
Flow Diagram of the Trial.

 By considering a mean difference (MD) of 3 and standard deviation (SD) on 3 the Parent and Teacher ADHD Rating Scale (based on pilot study), a power of 80%, and a two-tailed significance level of 0.05, the size of sample was determined at 23 patients for each group (n = 92). It is worth highlighting that based on Douglas and Montgomery, the minimum sample size required for studies with repeated measurement designs is 15 people.^24^ By predicting 10% attrition rate, 26 patients were finally calculated for each group (n = 104) (Figure 1).

###  Subjects

 The recruiting process started with the invitation of outpatients of both genders aged 6 to 17 years. The participants were screened for ADHD criteria according to the Diagnostic and Statistical Manual of Mental Disorders, Fifth Edition (DSM-5). First, a psychiatrist confirmed the diagnosis of ADHD according to the DSM-5 criteria for ADHD.^25,26^ In addition, the medical history of the patients was also collected. Moreover, reports of the parents and teachers were inspected carefully to measure the level of the children’s signs based on ADHD-RS-IV.

 The exclusion criteria were, other psychiatric disorders (except oppositional defiant disorder), score of intelligence quotient less than 70, serious medical circumstances like seizure, organic brain disorders, systolic blood pressure over 125 mm Hg and resting pulse under 60 or higher than 110 beats/min, and allergy to the D3 pearl. The final exclusion criterion was using any type of psychotropic drugs in the past two months. Additionally, before the initiation of any therapeutic activity, BMI, blood pressure and pulse rate were assessed.

###  Treatments

 A total of 120 participants were randomly divided in 4 parallel groups by block randomization, i.e. 30 participants in each block. The first, second and third experimental groups received D3 pearl, neurofeedback (Pro Comp) (30-min sessions 2 days a week) and combined D3 and neurofeedback, respectively. Our main hypothesis based on the literature and previous papers was for changes at electrode Cz for neurofeedback training(NFT). The vitamin D group received an oral supplementation (pearl) of 50 000 IU vitamin D3 (cholecalciferol) per week over 3 months (12 weeks). Additionally, blood samples were taken from children at baseline, 4, 8 and 12 weeks. Also, some general information like age and education, serum level of iron, total iron binding capacity, Ca and zinc were assessed at 4 steps of the trial in all patients. Moreover, serum amount of ferritin and 25-OH-D were assessed at baseline, 4, 8 and 12 weeks in all participants. The level of vitamin D was assessed based on serum 25-OH-D by enzyme-linked immunosorbent assay. Based on the level of vitamin D, deficiency was defined as follows: vitamin D inadequacy: 12 to 20 ng/mL (30 to 50 nmol/L); vitamin D deficiency: < 12 ng/mL (< 30 nmol/L).

###  Instrument

 Parent and Teacher Rating Scale-IV (ADHD-RS-IV) was utilized to rate the ADHD symptoms at baseline and after 4, 8, and 12 weeks.^25^ It is noticeable that this scale is extensively used in Iranian studies and offers high quality rating of attentive and behavioral disorders among school-aged children.^27^ Moreover, this scale measures the 18 obvious signs of ADHD according to a 4-point scale. In this study, the first measurement was conducted at baseline in each group. Other outcome measures were also conducted after 4, 8, and 12 weeks.

###  Statistical Analyses

 IBM SPSS 26 was used to analyze the obtained data. Number and percentage were used for presenting categorical variables. Also, mean (SD) was used for continuous variables and mean (95% confidence intervals, 95% CIs) was utilized to evaluate MD. For reporting and comparing ADHD Rating Scale scores across the patients, repeated measures ANOVA analysis and general linear model (GLM) were used. Noticeably, the type of analysis was intention-to-treat. Also, Levene’s test (0.971) confirmed the normality of variances. The potential confounders were adjusted at baseline. Besides, Greenhouse-Geisser correction was utilized for degrees of freedom. one-way measure analysis of variance with a two-tailed post hocdependent *t* test was conducted to assess the adequacy of these medications. To compare changes of scores from baseline between the participants, the *t* test was used. Also, Fisher’s exact test or the chi-square test were utilized for comparing categorical variables. In all steps of the analyses, a p-value < 0.05 was considered as the statistical significance level.

###  Safety

 It was explicitly mentioned that the participants were free to leave the trial on their authority and there was no obligation to continue. Moreover, they were informed to have an interview with the psychiatrist in case of side effects or similar problems due to the experiment. During the screening session, several aspects of their body were examined and registered including weight, vital signs like breathing, heartbeat and blood pressure. The other tests included hematology and serum chemistry. The patients were again requested to inform the researchers if they had any unexpected or unpleasant conditions. In addition, in all sessions, the psychiatrist checked their situation through checklists and the clinical interview.

## Results

 A total of 120 patients were screened in the present research. During the study, eight participants who did not meet the criteria were excluded. Finally, 112 patients were randomly allocated into four groups; 29 patients were randomly assigned to four groups, some of whom withdrew from the trial. Finally, 104 patients (26 participants in each block) finished the experiment (Figure 1). The baseline conditions of the participants are illustrated in Table 1.

**Table 1:** Baseline Characteristics of the Patients

**Variables**	**Groups**	**N**	**Mean**	**SD**
Age	Control	26	11.50	1.79
	Vitamin D	26	10.96	1.40
	Neurofeedback	26	11.62	2.08
	Neurofeedback and vitamin D	26	11.31	2.48
Parent inattention baseline	Control	26	19.85	1.59
	Vitamin D	26	20.58	2.34
	Neurofeedback	26	19.38	2.91
	Neurofeedback and vitamin D	26	19.54	3.62
Parent hyperactivity baseline	Control	26	19.73	1.59
	Vitamin D	26	20.08	1.47
	Neurofeedback	26	20.58	3.42
	Neurofeedback and vitamin D	26	18.85	3.07
Teacher inattention baseline	Control	26	19.54	1.39
	Vitamin D	26	19.92	2.02
	Neurofeedback	26	18.50	3.42
	Neurofeedback and vitamin D	26	19.19	3.27
Teacher hyperactivity baseline	Control	26	18.62	1.92
	Vitamin D	26	19.62	1.70
	Neurofeedback	26	18.19	2.58
	Neurofeedback and vitamin D	26	19.73	3.14
Parent total baseline	Control	26	39.58	2.45
	Vitamin D	26	40.65	2.23
	Neurofeedback	26	39.96	5.57
	Neurofeedback and vitamin D	26	38.38	6.18
Teacher total baseline	Control	26	38.15	2.52
	Vitamin D	26	39.54	2.47
	Neurofeedback	26	36.69	5.33
	Neurofeedback and vitamin D	26	38.92	5.91

N, number; SD, standard deviation.

###  Parent ADHD-RS-IV

**Figure 2:**
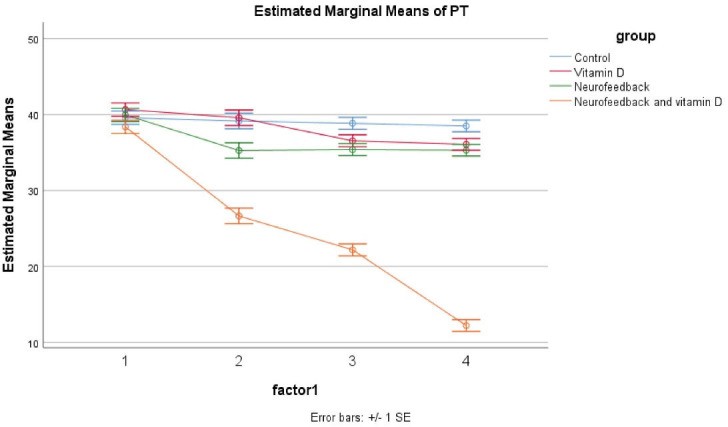
GLM Repeated Measure for Investigation of the Effects of Four Treatments.

 The results showed no significant difference regarding the parents scores at baseline in the control, vitamin D, neurofeedback, and neurofeedback and vitamin D groups. It was demonstrated by the GLM repeated measures that the trend of these four groups was not different across time as appearing within the impact of time × group interaction (Greenhouse–Geisser corrected: F = 0/970, *df* = 5, *P* = 0.505) (Figure 2). The same impacts were seen for the time × group interaction, and in the reduction of the group in hyperactivation and inattention subscales. Besides, a significant effect was observed for these four medications on decreasing ADHD symptoms (*P* < 0.05 for four groups); however, this effect was more significant in the combined group (*P* < 0.001 for these four groups). In the control, vitamin D, and neurofeedback groups, this effect was approximately similar and there were some fluctuations. In the combined group, there was a significant decrease in weeks 4, 8, and 12 (*P* < 0.05). Accordingly, this evaluation was also same for the Inattention and Hyperactive/Impulsive subscales (Table 2).

**Table 2:** Parents’ Rating of ADHD Symptoms in Participants by Week

**Group**s		**Week 4, ** **Mean (SD)**	**Mean difference (95% CI)**	***P***	**Week 8, ** **Mean (SD)**	**Mean difference (95% CI)**	***P***	**Week 12, Mean (SD)**	**Mean difference (95% CI)**	***P***
Parent Inattention	Control	19.46 (1.56)^a^	0.6 (18.86 –20.06)	0.667	19.65 (1.67)^a^	0.64 (20.29–19.01)	0.12	19.15 (2.13)^a^	0.82 (19.97–18.33)	0.222
	Vitamin D	19.62 (1.77)^a^	0.66 (18.96–20.28)	0.005	18.23 (2.34)^a^	0.90 (19.13–17.33)	0.003	18.15 (1.49)^a^	0.57 (18.72–16.66)	0.005
	Neurofeedback	17.65 (3.45)^a^	0.67 (18.97– 16.33)	0.004	17.62 (2.77)^a^	1.06 (18.68–16.65)	0.000	17.31 (2.22)^a^	0.85 (18.16–15.09)	0.000
	Neurofeedback and vitamin D	13.08 (3.94)^b^	1.51 (14.59–11.57)	0.002	11.04 (2.99)^b^	1.15 (12.55–10.25)	0.000	5.92 (2.58)^b^	0.99 (6.91–3.34)	0.000
Time effect		A			B			C		
Parent hyperactivity	Control	19.69 (1.67)^a^	0.64 (20.33–19.05)	0.698	19.19 (1.96)^a^	0.75 (19.94–18.44)	0.109	19.35 (1.67)^a^	0.64 (19.99–18.71)	0.336
	Vitamin D	19.96 (2.22)^ab^	0.43 (20.39–19.26)	0.006	18.31 (1.59)^a^	0.61 (18.92–17.7)	0.004	17.92 (1.65)^a^	0.63 (18.55–17.29)	0.000
	Neurofeedback	17.62 (3.65)^b^	0.71 (18.33–16.91)	0.002	17.77 (2.29)^a^	0.88 (18.65–16.89)	0.000	18 (2.43)^a^	0.93 (18.93–17.07)	0.000
	Neurofeedback and vitamin D	13.58 (3.83)^c^	0.75 (14.33–12.83)	0.000	11.15 (2.52)^b^	0.84 (11.99–10.31)	0.000	6.31 (3.54)^b^	1.36 (7.67–4.95)	0.002
Time effect		A			B			C		
Parent Total	Control	39.15 (2.71)^a^	0.53 (39.68–38.62)	0.239	38.85 (2.89)^a^	1.11 (39.96–37.74)	0.011	38.5 (3.18)^a^	1.22 (39.72–37.28)	0.456
	Vitamin D	39.58 (2.1)^a^	0.39 (39.97–39.19)	0.000	36.54 (3.08)^a^	1.18 (37.72–35.36)	0.000	36.08 (2.59)^b^	0.99 (37.07–35.09)	0.006
	Neurofeedback	35.27 (6.91)^b^	1.35 (36.62–33.92)	0.0000	35.38 (4.71)^a^	1.81 (37.19–33.57)	0.000	35.31 (3.84)^b^	1.47 (36.78–33.84)	0.000
	Neurofeedback and vitamin D	26.65 (6.92)^c^	1.359 (28–25.29)	0.000	22.19 (4.97)^b^	1.91 (24.1–20.28)	0.000	12.23 (5.49)^c^	2.11 (14.34–10.12)	0.000
Time effect		A			B			C		

The lowercase letters indicate the comparison of the groups and the capital letters are for comparison. Groups that have common letters do not have significant differences, while groups that do not have common letters have significant differences.

###  Teacher ADHD-RS-IV

**Figure 3:**
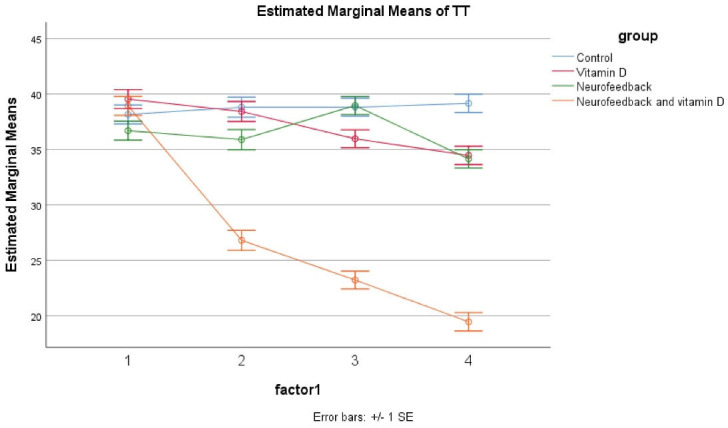
GLM Repeated Measure for Investigation of the Effects of Four Treatments.

 The results showed no significant differences at baseline across the four groups including control, vitamin D, neurofeedback, and neurofeedback and vitamin D. The GLM repeated measures showed that the trend of the four groups was not different during time as observed in the effect of time × group interaction (Greenhouse– Geisser corrected: F = 0.976, *df* = 5, *P* = 0.601 601) (Figure 3). Also, the same impacts were seen for the time × group interaction, and in the reduction of the group in hyperactivation and inattention subscales of the Teacher ADHD-RS-IV. Besides, significant effects of these four medications were illustrated on improving Teachers Rating (*P* < 0.05 for four groups); however, the effect of the combined group was more significant (*P* < 0.05 for these four groups). In the control, vitamin D and neurofeedback, and neurofeedback groups, this effect was approximately similar and there were also some fluctuations. In the combined group, post hoc comparisons illustrated a significant decrease after 4, 8, and 12 weeks (*P* < 0.05). This evaluation was the same for the inattention and hyperactive/impulsive subscales (Table 3).

**Table 3:** Parents’ Rating of ADHD Symptoms in Participants by Week

**Groups**		**Week 4, ** **Mean (SD)**	**Mean Difference (95% CI)**	***P***	**Week 8, ** **Mean (SD)**	**Mean Difference (95% CI)**	***P***	**Week 12, Mean (SD)**	**Mean Difference (95% CI)**	***P***
Teacher inattention	Control	19.31 (1.89)^a^	0.72 (20.03–18.59)	0.567	19.23 (1.73)^a^	0.66 (19.89–18.57)	0.203	19.46 (1.68)^a^	0.64 (20.1–18.82)	0.714
	Vitamin D	19.27 (2.07)^a^	0.79 (20.06–18.48)	0.008	18.04 (2.22)^ab^	0.85 (18.89–17.19)	0.006	17.38 (2.04)^b^	0.78 (18.16–16.6)	0.007
	Neurofeedback	18.15 (3.17)^a^	1.22 (9.37–16.93)	0.006	19.23 (1.7)^b^	0.65 (19.88–18.58)	0.003	17.15 (2.2)^b^	0.84 (17.99–16.31)	0.003
	Neurofeedback and vitamin D	13.15 (2.95)^b^	1.13 (14.28–12.02)	0.000	11.5 (2.61)^c^	1 (12.5–10.5)	0.003	9.19 (3.33)^c^	1.28 (10.47–7.91)	0.001
Time effect		A			A			B		
Teacher hyperactivity	Control	19.5 (1.84)^a^	0.70 (20.02–18.08)	0.101	19.58 (1.53)^a^	0.58 (20.16–19)	0.312	19.69 (2)^a^	0.77 (20.46–18.92)	0.459
	Vitamin D	19.15 (1.38)^a^	0.53 (19.68–18.62)	0.004	17.92 (3.05)^ab^	1.17 (19.09–16.75)	0.007	17.08 (2.58)^b^	0.99 (18.07–16.09)	0.000
	Neurofeedback	17.73 (3.54)^a^	0.38 (18.11–17.35)	0.004	19.73 (2.16)^b^	0.83 (20.56–18.9)	0.003	17 (2.42)^b^	0.92 (17.92–16.8)	0.000
	Neurofeedback and vitamin D	13.65 (2.46)^b^	0.94 (14.59–12.71)	0.000	11.73 (2.92)^c^	1.12 (12.85–10.61)	0.000	10.27 (3)^c^	1.29 (18.87–16.49)	0.000
Time effect		A			A			B		
Teacher Total	Control	38.81 (3.03)^a^	1.16 (39.97–37.65)	0.604	38.81 (2.87)^a^	1.1 (39.91–37.71)	0.015	39.15 (2.94)^a^	1.13 (40.28–38.02)	0.000
	Vitamin D	38.42 (2.86)^ab^	1.1 (39.52–37.32)	0.005	35.96 (4.52)^ab^	1.74 (37.7–34.22)	0.004	34.46 (4.09)^a^	1.54 (36– 39.92)	0.000
	Neurofeedback	35.88 (6.56)^b^	2.52 (38.4–33.36)	0.003	38.96 (3.39)^b^	1.3 (40.26–37.66)	0.000	34.15 (4.24)^a^	1.63 (35.78–32.52)	0.000
	Neurofeedback and vitamin D	26.81 (4.49)^c^	1.72 (28.53–25.09)	0.001	23.23 (5.19)^c^	1.99 (25.22–21.24)	0.000	19.46 (5.26)^a^	2.02 (21.48–17.44)	0.000
Time effect		A			A			B		

The lowercase letters indicate the comparison of the groups and the capital letters are for comparison. Groups that have common letters do not have significant differences, while groups that do not have common letters have significant differences.

###  Side Effects

 Common side effects which have the highest frequency were reported. No dangerous side effect was reported in any of the patients during 12 weeks. Moreover, fortunately, all the reported side effects ranged from mild to moderate. The rate of reported side effects was not different across all the study groups for 12 weeks.

## Discussion

 The outcomes of the trial present evidence for the effectiveness of the combined neurofeedback with vitamin D supplementation in children with ADHD compared to vitamin D and neurofeedback alone. Moreover, parents and teachers scores improved considerably in patients of the combined group. Also, in the vitamin D and neurofeedback groups, a reduction was reported. The novel point of this study was that combined neurofeedback with vitamin D supplementation is more effective than other interventions and this intervention even could reduce the duration of therapy to 4 weeks. The difference between the combined intervention and other interventions in this trial is so obvious that the effect of the combined intervention can be easily inferred (Figures 2 and 3).

 The clinical importance of these outcomes was supported by the reported assessments in both raters. Since ADHD is complex and a variety of underlying factors have been implicated, such as decreased fast brain waves in the prefrontal cortex, cerebellum, and basal ganglia,^28,29^ it is necessary to search for proven treatments. Some studies have shown severe side effects of routine treatments such as low appetite, abdominal pain, weight loss, insomnia, dizziness, dry mouth, nervousness, emotional lability and headache. Although it is rare, more severe side effects of these medications have been reported such as psychosis and seizures.^30,31^ Therefore, there is a growing tendency toward treatments with low side effects including neurofeedback and some supplementation.

 Nutrition has an obvious place in the case of ADHD and reappeared during the last recent years. The prominent topics in this field are the diets, fast foods, utilizing low level of minerals and lack of consuming fruits and vegetables. Moreover, an association between food deficits and ADHD symptom severity has been reported.^32,33^

 These findings together are assumed as potentially important components that may affect related disorders and the need for more nutrients than what might be found in the common food basket. The availability of some or all these components could significantly decrease the availability of nutrients for optimized neural system health. In addition, by taking all these components into account, supplementation might be considered than the manipulation of diet in patient with ADHD.^34^

 So far, the remedy of ADHD using a single nutrient or one-aspect approach has yielded findings that are unreliable and contradictory.^35^ “A set of components also supports the multi-nutrient hypothesis that seems valuable to be investigated. Considering the physiological aspect, there are several nutrients which are necessary for the biological process, as it has been proven that they contribute to the methylation cycle and Krebs cycle. Also, it could be a remarkable idea to combine nutrients to improve the metabolic performance”.^34^

 Some evidence supports the effectiveness of vitamin D in the regulation of the synthesis of serotonin by tryptophan hydroxylase 2 enzymes.^36^ Furthermore, vitamin D also has an important role in adjusting the signaling of calcium (It is notable that high levels of Ca ions lead to toxicity. Vitamin D helps to avoid this phenomenon by diluting the high level).^37^ Vitamin D deficiency was also shown to be related to difficulties in cognition, although the underlying mechanisms are not completely elucidated. The extracellular matrix (ECM) appears as an effective factor in the plasticity of the synapse and a novel assumption is that Vitamin D may interact with aggregates of the ECM and peri-neuronal nets (PNNs), in regulation of brain system plasticity. So, dysfunctional performance of PNNs resulting from vitamin D deficiency may play a role in the development of mental conditions.^37^ Based on the evidence, synaptic plasticity is hypothesized to be an obvious and explicit processes mediating the functions like memorizing and learning.^38^ In addition, the role of vitamin D in mediating long-term potentiation (LTP) in ADHD has recognized the mechanism of synaptic plasticity and a key factor in data retention in the brain. Furthermore, of particular note, LTP is completely related to calcium (Ca^2 +^) rise in the postsynaptic cell, through voltage-gated calcium channels or N-methyl-D-aspartate receptors.^38,39^

 On the other side, the effectiveness of neurofeedback is dependent on the executive functions including the performance of working memory network and right middle frontal and inferior frontal regions.^40^ Also, all protocols of neurofeedback could be used for performance promotion in a variety of neurological disorders linked to abnormal brain connectivity.^41^ Moreover, the stability of theta waves in the frontal cortex (F3) is associated with distraction and inattention, so neurofeedback is likely to improve the symptoms of the disease by helping the regulation of these waves and the activity of the frontal lobe. On the other hand, since the ability to change from one mental state or a cognitive process to another state or another cognitive process is mainly implemented by anterior cingulate gyrus and given the proximity of this anatomical region to the frontal cortex, it seems logical that neurofeedback training can be effective on this area. Based on the available evidence stating that the ratio of theta to beta waves in children is significantly higher than the pattern of the ratio of these waves in non-volatile individuals, therefore, neurofeedback protocols with the aim of suppressing theta and strengthening beta or low frequency beta have high therapeutic values in patient with ADHD. The reason for the effectiveness of neurofeedback-based intervention can be the increased activity of the anterior cingulate cortex region, which has specific importance in modulating cognitive and emotional processes in the brain. Electroencephalographic studies indicate a negative relationship between anterior cingulate cortical region activity and theta strength as well as a positive relationship between beta power and frontal medial activity. As a result, by suppressing theta and simultaneously increasing the beta, an increase can be observed in the activity of the frontal intermediate areas, especially in the dorsal part of the anterior cingulate, which is essentially low functioning in the children with ADHD. This increase in activity improves the symptoms of attention in these children as well.^42^

 In the combined intervention group, it seems that the integrative effect of improving brain connectivity, improving executive functions, and regulation of theta waves with regulating the synthesis of serotonin the signaling of calcium lead to the decrease of ADHD symptoms. Accordingly, one of the reasons for this influence must be sought in the physiological dimension. Psychologically, in the combined treatments, it is generally believed that VDR exist in neurons and glial cells in many regions of the neural system such as the cortex, deep grey matter, cerebellum, brainstem nuclei, substantia nigra (dopaminergic neuron-rich area), spinal cord, and ventricular system.^43^ Also, a relation between vitamin D and PNNs has been proposed by a quite new hypothesis aggregate the structures of the ECM.^38^

 Although the current trial had various benefits such as randomization design and double blinding with a control group, there also were some limitations. Lack of follow-up should also be considered in this regard. In addition, future placebo-controlled trials and cross-over trials using concrete neuroimaging instruments, as well as in-depth information such as clinical interviews could be excellent options for performing future studies.

 The results of this study revealed some evidence related to the effectiveness of combined treatments. The final findings showed that the patients in the third experimental group experienced greater reduction in term of symptoms compared to the first and second experimental groups. To the best of our knowledge, the combined treatment could be more effective than each treatment alone. Since the response to all stimulants in patients with ADHD is less than 30% and based on laboratory evidence, these drugs have serious side effects, the outcomes of the present trial present a novel efficacious therapeutic option for this disorder. Nevertheless, considering the need for filling research gaps, different studies in other regions should be performed in the future.
